# Transmissibility of COVID-19 in 11 major cities in China and its association with temperature and humidity in Beijing, Shanghai, Guangzhou, and Chengdu

**DOI:** 10.1186/s40249-020-00708-0

**Published:** 2020-07-10

**Authors:** Xiao-Jing Guo, Hui Zhang, Yi-Ping Zeng

**Affiliations:** Institute of Public Safety Research, Tsinghua University, Beijing, 100084 People’s Republic of China

**Keywords:** COVID-19, Transmissibility, Basic reproduction number, Temperature, Humidity

## Abstract

**Background:**

The new coronavirus disease COVID-19 began in December 2019 and has spread rapidly by human-to-human transmission. This study evaluated the transmissibility of the infectious disease and analyzed its association with temperature and humidity to study the propagation pattern of COVID-19.

**Methods:**

In this study, we revised the reported data in Wuhan based on several assumptions to estimate the actual number of confirmed cases considering that perhaps not all cases could be detected and reported in the complex situation there. Then we used the equation derived from the Susceptible-Exposed-Infectious-Recovered (SEIR) model to calculate *R*_0_ from January 24, 2020 to February 13, 2020 in 11 major cities in China for comparison. With the calculation results, we conducted correlation analysis and regression analysis between *R*_0_ and temperature and humidity for four major cities in China to see the association between the transmissibility of COVID-19 and the weather variables.

**Results:**

It was estimated that the cumulative number of confirmed cases had exceeded 45 000 by February 13, 2020 in Wuhan. The average *R*_0_ in Wuhan was 2.7, significantly higher than those in other cities ranging from 1.8 to 2.4. The inflection points in the cities outside Hubei Province were between January 30, 2020 and February 3, 2020, while there had not been an obvious downward trend of *R*_0_ in Wuhan. *R*_0_ negatively correlated with both temperature and humidity, which was significant at the 0.01 level.

**Conclusions:**

The transmissibility of COVID-19 was strong and importance should be attached to the intervention of its transmission especially in Wuhan. According to the correlation between *R*_0_ and weather, the spread of disease will be suppressed as the weather warms.

## Background

On December 8, 2019, the first case of unexplained pneumonia was officially reported in Wuhan, the capital of Hubei Province in China [1]. There have been reports of the new coronavirus disease (coronavirus disease 2019, COVID-19, named by the World Health Organization on February 11, 2020) since December 2019 [1, 2]. As was reported by the National Health Commission of the People’s Republic of China, the number of confirmed cases had reached 63 851 by February 13, 2020 in China, including 1380 deaths. On the same day, Hubei Province alone totally had 51 986 confirmed cases including 1318 deaths, accounting for 81.4% and 95.5% of the whole country respectively. Among them there were 35 991 confirmed cases and 1016 deaths in Wuhan, accounting for 69.2% and 77.1% of the number in Hubei Province respectively [3]. The cumulative number of confirmed cases keeps rising, indicating the strong transmissibility of COVID-19, especially in Wuhan, Hubei Province. Therefore, it is of great importance to adopt reasonable indicators to assess the transmission ability of the disease, based on which effective intervention and control measures could be put forward [4, 5].

The basic reproduction number (*R*_0_) refers to the expected number of cases generated from a single case when all people are susceptible to infection [6]. It is widely used to evaluate the transmission ability of an emerging infectious disease and determine what degree of control measures should be taken to eradicate the disease [7–10]. When *R*_0_>1, the disease starts to spread; and when *R*_0_<1, the disease is effectively controlled [11]. *R*_0_ is influenced by many other factors except for the characteristics of the disease itself, such as conditions of the environment, policies of the government, people’s awareness of infectious diseases, and social behavior. Therefore, we can use *R*_0_ to measure the transmissibility of COVID-19 and analyze its influencing factors, which provides data support for suggestion-proposing and decision-making.

Research on transmissible diseases like influenza [12], severe acute respiratory syndrome (SARS) [13] and Middle East respiratory syndrome (MERS) [14] has found that disease transmission is associated with temperature and humidity of the environment [15–20]. In terms of biological methods, influenza virus spread was found to be promoted by cold temperature and low relative humidity with the guinea pig as a model host [21]; besides, an experiment on the SARS coronavirus indicated that high temperature and high humidity suppressed the spread of the virus [22]; similarly, MERS coronavirus was more stable when temperature or humidity was lower [23]. In terms of statistical methods, case studies of SARS in four major cities in China suggested that the transmissibility had a close relationship with temperature and its variation [24]; and a regression equation was derived to show how temperature, relative humidity, and wind velocity affected the transmission of SARS [25]. Thus we wonder if the spread of COVID-19 follows a similar pattern. Considering that *R*_0_ is useful for measuring the transmission ability of infectious diseases, we conducted association analyses between *R*_0_ and temperature, relative humidity, and absolute humidity respectively. Statistical methods such as correlation and regression were adopted for the analysis.

This paper measured the transmissibility of COVID-19 with *R*_0_ and analyzed its correlation with temperature and humidity. First, we revised the epidemiological data in Wuhan to make *R*_0_ more accurate. Second, we calculated *R*_0_ and compared the average value and developing trend of *R*_0_ in 11 cities including Wuhan. Third, we conducted correlation and regression analysis between *R*_0_ and temperature and humidity to see the association between *R*_0_ and weather.

## Methods

### Data acquisition and preprocessing

The daily accumulative number of confirmed cases and new additions are reported by the National Health Commission of the People’s Republic of China as well as the health commission of each province on the official website. An R package has been developed to access the epidemiological data directly [26]. The R package was used by us to acquire the number of total cases and new additions from January 18, 2020 to February 13, 2020 in Wuhan, Hubei Province considering that the situation there was complex and needed much attention. Besides, we also collected the daily-reported accumulative number of confirmed cases from January 24, 2020 to February 13, 2020 in 10 Chinese major cities outside Hubei Province including Beijing, Chengdu, Chongqing, Guangzhou, Hangzhou, Hefei, Nanjing, Shanghai, Shenzhen, and Zhengzhou (listed by initials) for further calculation, estimation, and analysis. The reasons for selecting those 10 cities were that they were first-tier cities or capital cities in China with the top number of cases. Certainly, Wuhan also met the criteria. Those cities could well represent the process status of the disease based on which disposal measures could be put forward.

As for Wuhan, it was estimated by Imperial College London, UK that the total number of confirmed diagnoses had reached 4000 by January 18, 2020 [27], which was much higher than the officially reported number. So we attempted to revise the data in Wuhan to infer the actual transmissibility of the new coronavirus. With the substantial enhancement of case detection and reporting, the differences between the official numbers and the estimates are predicted to be fewer and fewer. There are several assumptions for the data-preprocessing procedure:

1. The first case appeared on December 8, 2019 in Wuhan and transmission started from that day on [1, 28].
2. The cumulative number of cases *Y*(*t*) by day *t* since the first single case followed the exponential function *Y*(*t*)=*e*^*λt*^ in early development [29].
3. The cumulative number of cases on January 18, 2020 was 4000, that was, *Y*(41)=4000 [27].
4. From February 13, 2020 on, all cases in Wuhan can be confirmed and the number of daily new cases is correct, given that the number of newly confirmed diagnoses on February 12, 2020 in Wuhan increased significantly, exceeding 10 000.

Based on those assumptions, the data-revising procedure in Wuhan is as follows:

1. According to assumption 2 and 3, the exponential growth rate is estimated as *λ*=*ln*[*Y*(41)]/41.
2. According to assumption 2 and 3, the number of new additions on January 18, 2020 equals *Y*(41)−*Y*(40)=4000−*e*^*λ*∗40^=733.
3. According to assumption 4, the number of new additions on February 13, 2020 is 2997, which is consistent with the officially reported number.
4. According to assumption 2, the daily number of new additions *y*(*t*) can be calculated by
  1 $$\begin{array}{@{}rcl@{}} y(t)&=&Y(t)-Y(t-1)\\ &=&e^{\lambda t}-e^{\lambda (t-1)}\\ &=&e^{\lambda t}\left(1-e^{-\lambda}\right). \end{array} $$
  Thus
  2 $$\begin{array}{@{}rcl@{}} ln[y(t)]=ln\left(1-e^{-\lambda}\right)+\lambda t. \end{array} $$
  So the relationship between *ln*[*y*(*t*)] and *t* is linear. Replace *ln*(1−*e*^−*λ*^) with *a* and *λ* with *b* in Eq. (2), and the coefficients *a* and *b* of the linear equation can be determined by substituting *y*(41)=733 and *y*(67)=2997 into the equation respectively.
5. The number of new additions each day from January 19, 2020 to February 12, 2020 can be calculated through the equation *y*(*t*)=*e*^*a*+*bt*^, where *a* and *b* are the coefficients obtained in procedure 4.
6. With the daily number of new additions known, the daily cumulative number of cases from January 19, 2020 to February 13, 2020 can be calculated by *Y*(*t*)=*Y*(*t*−1)+*y*(*t*),*t*=42,43,...,67.

As for other cities outside Hubei Province, it is assumed that the officially reported data is accurate. Based on the relationship *ln*[*Y*(*t*)]=*λt*, we performed logarithmic fitting between the cumulative number of diagnoses and time and inferred that transmission started on December 27, 2019 outside Hubei Province.

### Calculation of the basic reproduction number

The basic reproduction number indicates the average number of people infected by a patient during the infectious period in the absence of control interventions [6]. It is also denoted *R*_0_, which measures the transmissibility of infectious diseases. There are several ways to estimate *R*_0_, including formula derivation [30, 31] and model fitting [32–34].

We describe the transmission pattern of COVID-19 with the Susceptible-Exposed-Infectious-Recovered (SEIR) model. In the exposed stage, an individual infection is not able to infect others. The duration of the exposed stage *T*_*E*_ is also called the latent period. While in the infectious stage with a duration of *T*_*I*_, an infected person does infect susceptible people. Assuming that the cumulative number of confirmed diagnoses increases exponentially in the early stages of an epidemic, the relationship between the basic reproduction number *R*_0_ and the exponential growth rate *λ* can be written as [35].

3 $$\begin{array}{@{}rcl@{}} R_{0} = \left(1+\lambda T_{E}\right)\left(1+\lambda T_{I}\right). \end{array} $$

The serial interval *T*_*g*_ is the sum of *T*_*E*_ and *T*_*I*_. Let *f*=*T*_*E*_/*T*_*g*_ be the ratio of the latent period to the serial interval, and then the basic reproduction number can be expressed as [29].

4 $$\begin{array}{@{}rcl@{}} R_{0} &=& 1+\lambda\left(T_{E}+T_{I}\right)+\lambda^{2}T_{E}T_{I}\\ &=& 1+\lambda T_{g}+\lambda^{2}T_{E}\left(T_{g}-T_{E}\right)\\ &=& 1+\lambda T_{g}+\lambda^{2}fT_{g}\left(T_{g}-fT_{g}\right)\\ &=& 1+\lambda T_{g}+f(1-f)\left(\lambda T_{g}\right)^{2}. \end{array} $$

The exponential growth rate is *λ*=*ln*[*Y*(*t*)]/*t*, where *t* is the number of days required to generate the cumulative number of *Y*(*t*) cases from the first case. According to the research on the first 425 patients with confirmed COVID-19, the mean latent period *T*_*E*_ = 5.2 (days) and the mean serial interval *T*_*g*_ = 7.5 (days) [36]. Adopting these values, we can calculate the ratio of the latent period to the serial interval by *f*=*T*_*E*_/*T*_*g*_=5.2/7.5=0.69.

### Correlation and regression analysis between *R*_0_ and weather

Correlation analysis is a commonly used statistical method to study the relationship between variables [37]. Regression analysis determines the quantitative relationship between two variables in statistics [38]. Among all kinds of regression methods, linear regression establishes the relationship between the dependent variable *Y* and the independent variable *X* with a linear equation *Y*=*a*+*bX* [39]. There are two coefficients in the equation, *a* as the intercept and *b* as the slope. We performed correlation analysis and linear regression between *R*_0_ and weather variables with the statistical analysis software IBM SPSS Statistics 25. The procedure is listed below.

1. We collected the data of the daily average temperature and relative humidity from January 24, 2020 to February 13, 2020 in four Chinese major cities which were Beijing (the capital of China), Shanghai (the municipality of China), Guangzhou (the capital of Guangdong Province) and Chengdu (the capital of Sichuan Province). We calculated absolute humidity from the temperature and relative humidity.
2. We imported the data of temperature, relative humidity, and absolute humidity together with *R*_0_ into the SPSS software and added cities as the classification label.
3. Through correlation analysis, the Pearson correlation coefficients between *R*_0_ and temperature, relative humidity, and absolute humidity were calculated respectively.
4. Through regression analysis, the intercept *a* and the slope *b* of the linear equation were estimated with *R*_0_ as the dependent variable *Y* and temperature, relative humidity or absolute humidity as the independent variable *X*.
5. We split the data by the city label and repeated procedure 3 and 4 for each city separately.

### Sensitivity analysis of *R*_0_

To analyze the sensitivity of *R*_0_ to the three key parameters in Eq. (4): *R*_0_=1+*λT*_*g*_+*f*(1−*f*)(*λT*_*g*_)^2^, we differentiated *R*_0_ to *λ*, *T*_*g*_ and *f* respectively:

5 $$\begin{array}{@{}rcl@{}} \frac{\partial R_{0}}{\partial \lambda} &=& T_{g} + 2f\left(1-f\right)\lambda T_{g}^{2}, \end{array} $$

6) (7 $$\begin{array}{@{}rcl@{}} \frac{\partial R_{0}}{\partial T_{g}}&=& \lambda + 2f\left(1-f\right)\lambda^{2} T_{g},\\ \frac{\partial R_{0}}{\partial f}&=&\left(1-2f\right)\left(\lambda T_{g}\right)^{2}. \end{array} $$

The sensitivity of the basic reproduction number *R*_0_ to the exponential growth rate *λ*, the serial interval *T*_*g*_, and the latent period ratio *f* can be estimated according to the range of variables and the scale of partial derivatives.

## Results

### Comparisons of transmission among different cities

**Fig. 1:**
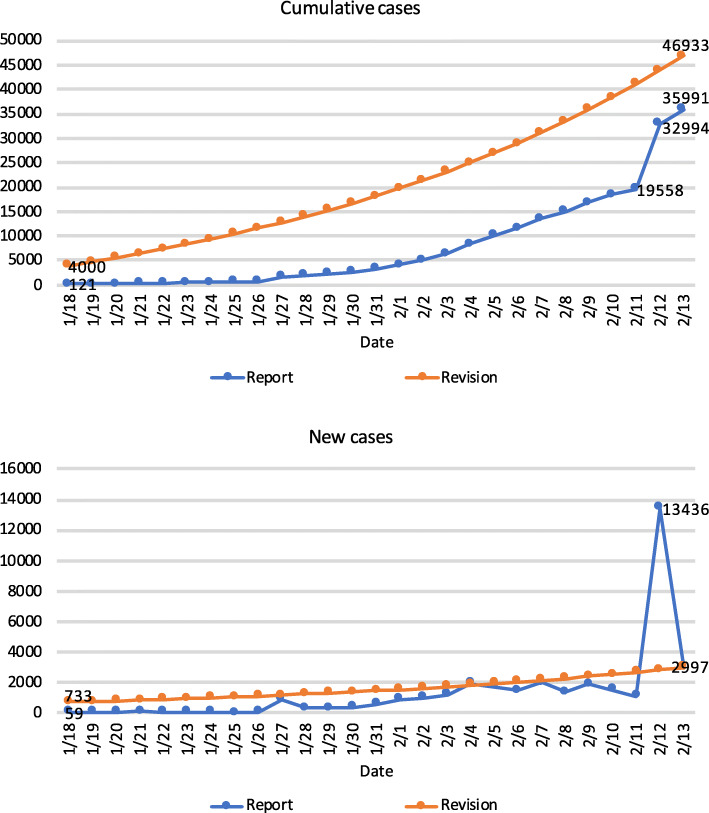
Comparisons between reported and revised data in Wuhan

The comparison between officially reported data and revised data in Wuhan is presented in Fig. 1 with important points marked on it. The estimated number of cumulative cases was higher than the official number every day, and it had reached 46 933 by February 13, 2020, which was 1.3 times that of the official number 35 991. The unusual high peak of new cases on February 12, 2020 was smoothed by revision.

**Fig. 2:**
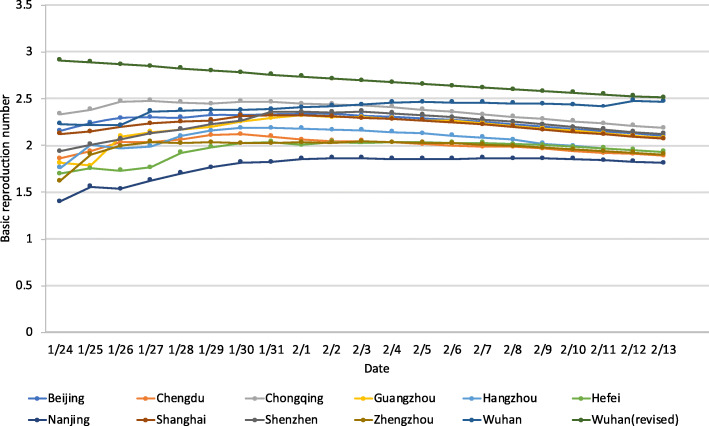
Calculation results of the basic reproduction number

The calculation results of the basic reproduction number *R*_0_ from January 24, 2020 to February 13, 2020 in 11 Chinese major cities are shown in Fig. 2. The values with the label “Wuhan” were calculated using the officially reported number of cases, while those with “Wuhan (revised)” were calculated using the revised number of cases. In this way, the broken line of “Wuhan” reflects the changing trend of *R*_0_, and the one of “Wuhan (revised)” reflects the value size of *R*_0_. It is assumed that the cumulative number of confirmed cases reported officially in cities outside Hubei Province is accurate, so the broken lines of the other 10 cities represent not only trends but also actual values.

As can be seen from Fig. 2, *R*_0_ in Wuhan is significantly higher than those in cities outside Hubei Province. Besides, *R*_0_ in cities outside Hubei Province has begun to decrease, while *R*_0_ in Wuhan does not show a significant downward trend.

For a more detailed analysis, the average basic reproduction number of the 21 days in each city and the date of the inflection point are presented in Table 1. The cities are listed by the average *R*_0_ from high to low. The inflection point refers to the day after which *R*_0_ shows a downward trend.

**Table 1:** The average *R*_0_ and the inflection point of each city (listed by the average *R*_0_)

City	Average *R*_0_	Inflection point
Wuhan	2.7	None
Chongqing	2.4	1/30
Beijing	2.3	2/2
Shenzhen	2.2	2/3
Shanghai	2.2	2/1
Guangzhou	2.2	2/1
Hangzhou	2.1	1/31
Chengdu	2.0	1/30
Zhengzhou	2.0	2/3
Hefei	2.0	2/2
Nanjing	1.8	2/2

It can be seen from Table 1 that the average *R*_0_ in Wuhan far exceeds those in other cities, which is 0.3 higher than that in Chongqing, the city which ranks second. It should be noted that the average *R*_0_ in Wuhan is calculated with the revised data to better fit the real value. In fact, the average basic reproduction number calculated with the officially reported data is also much higher than those in other cities, which is 2.4.

The inflection points of cities outside Hubei Province range from January 30 to February 3, while the inflection point of Wuhan had not appeared because the number of confirmed cases had kept increasing rapidly by February 13, 2020. Although *R*_0_ in Wuhan reaches a peak on February 12, it cannot be determined that February 12 is the inflection point. Because since that day, Hubei Province has included the number of clinically diagnosed cases into the number of confirmed cases. The modification of the diagnostic criteria leads to a sudden increase of newly confirmed patients, which explains why *R*_0_ is particularly high on February 12.

### Correlation between *R*_0_ and temperature

The Pearson correlation coefficients and significance between *R*_0_ and temperature are shown in Table 2. The row of “Summary” suggests that calculated as a whole, the correlation between *R*_0_ and temperature is statistically significant at the 0.01 level. The correlation coefficient is -0.459, so *R*_0_ and temperature have a negative correlation, which means that *R*_0_ decreases as the temperature increases. The higher the temperature, the lower the transmission capability. As for the analysis of each city, *R*_0_ negatively correlates with temperature in Shanghai and Chengdu, correlation significant at the 0.01 level. Correlation is not significant in Beijing and Guangzhou. Over the study period, the average *R*_0_ in Beijing, Shanghai, Guangzhou, and Chengdu are 2.3, 2.2, 2.2, and 2.0 respectively and the average temperatures are -1.0 ^∘^C, 7.9 ^∘^C, 14.9 ^∘^C, and 9.9 ^∘^C respectively. There is not a significant relationship between the average *R*_0_ in a city versus its average temperature (*r*=−0.486, *P*>0.5).

**Table 2:** Correlation analysis between *R*_0_ and temperature

	Pearson correlation	Significance (2-tailed)	n
Summary	-0.459 ^∗∗^	0.000	84
Beijing	-0.429	0.052	21
Shanghai	-0.735 ^∗∗^	0.000	21
Guangzhou	-0.410	0.065	21
Chengdu	-0.732 ^∗∗^	0.000	21

^∗∗^Correlation is significant at the 0.01 level (2-tailed)

Linear regression was performed on the data for all cities combined as well as the data in Shanghai and Chengdu which showed a significant correlation. Table 3 presents the linear regression results. Replace *a* and *b* in the equation *R*_0_=*a*+*bT* (where *T* is temperature) with the corresponding actual values in Table 3, and correlation between *R*_0_ and temperature can be expressed more precisely. For example, the linear regression equation of Shanghai is written as *R*_0_=2.424−0.026*T*. It can be inferred from *b*<0 that *R*_0_ negatively correlates with temperature in Shanghai, which is consistent with the correlation analysis result above.

**Table 3:** Linear regression analysis of temperature to *R*_0_

	*a*	Std. error of *a*	*b*	Std. error of *b*
Summary	2.240	0.021	-0.010	0.002
Shanghai	2.424	0.045	-0.026	0.006
Chengdu	2.259	0.056	-0.026	0.006

**Fig. 3:**
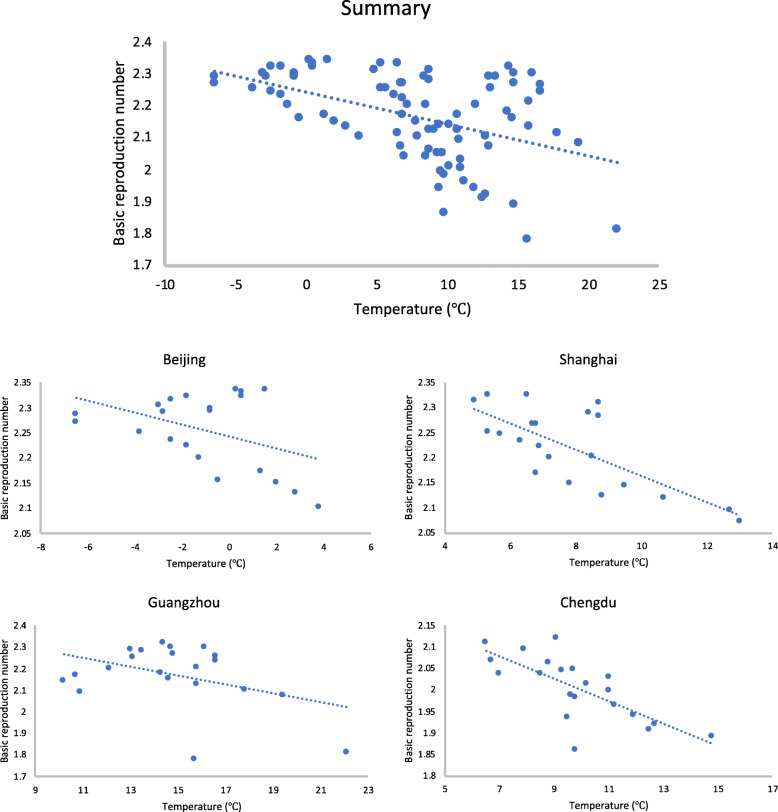
Scatter plot of temperature and basic reproduction number

We plotted every pair of temperature and *R*_0_ in a city or the whole data on the scatter figure to make correlation more intuitive, which was presented in Fig. 3. The regression lines followed the corresponding linear regression equations.

### Correlation between *R*_0_ and relative humidity

The Pearson correlation coefficients and significance between *R*_0_ and relative humidity are presented in Table 4. According to the first row, the correlation between *R*_0_ and relative humidity is statistically significant at the 0.01 level in general. The correlation coefficient is -0.391, indicating that *R*_0_ decreases as the relative humidity increases. As for the analysis of each city, *R*_0_ negatively correlates with relative humidity in Beijing and Shanghai, which is significant at the 0.01 level. While the correlation is significantly positive in Chengdu at the 0.01 level, which implies that the transmission ability and relative humidity have consistent trends there. Correlation is not significant in Guangzhou.

**Table 4:** Correlation analysis between *R*_0_ and relative humidity

	Pearson correlation	Significance (2-tailed)	*n*
Summary	-0.391 ^∗∗^	0.000	84
Beijing	-0.568 ^∗∗^	0.007	21
Shanghai	-0.722 ^∗∗^	0.000	21
Guangzhou	-0.363	0.106	21
Chengdu	0.619 ^∗∗^	0.003	21

^∗∗^Correlation is significant at the 0.01 level (2-tailed)

The correlation was significant in Beijing, Shanghai, and Chengdu, and thus we conducted linear regression on the data of the three cities as well as the summary of all cities. The linear regression results are presented in Table 5. Replace *a* and *b* in the equation *R*_0_=*a*+*bRH* (where *RH* is relative humidity) with the corresponding actual values in Table 5, and the correlation between *R*_0_ and relative humidity can be expressed with a quantitative method.

**Table 5:** Linear regression analysis of relative humidity to *R*_0_

	*a*	Std. error of *a*	*b*	Std. error of *b*
Summary	2.415	0.067	-0.004	0.001
Beijing	2.417	0.056	-0.003	0.001
Shanghai	2.542	0.072	-0.004	0.001
Chengdu	1.651	0.103	0.005	0.001

**Fig. 4:**
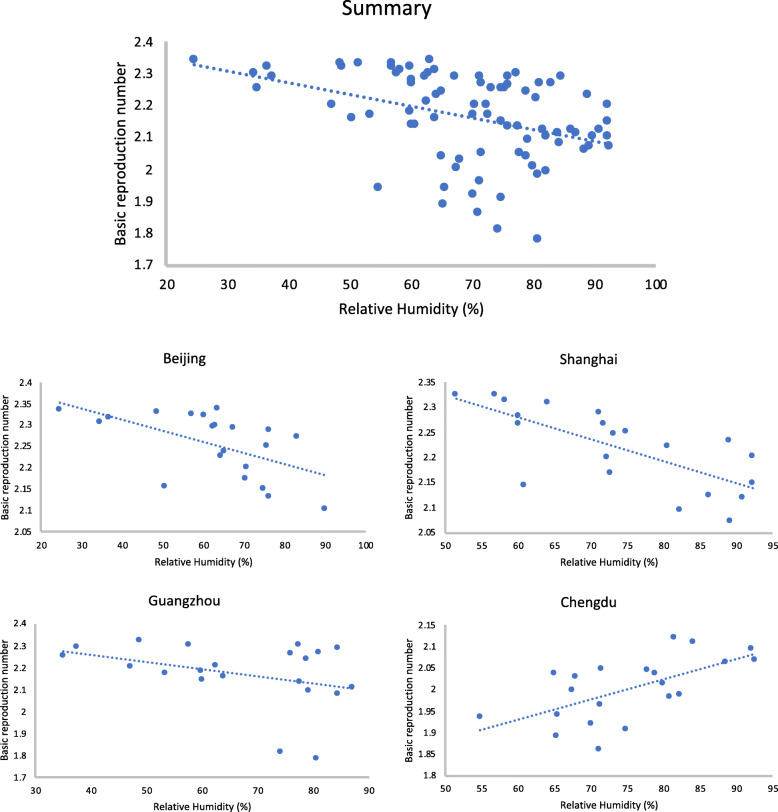
Scatter plot of relative humidity and basic reproduction number

The scatterplots and corresponding regression lines of relative humidity and *R*_0_ summarized across all cities and by individual cities are presented in Fig. 4.

### Correlation between *R*_0_ and absolute humidity

The Pearson correlation coefficients and significance between *R*_0_ and absolute humidity are presented in Table 6. The negative correlation between *R*_0_ and absolute humidity is significant in general as well as in Beijing, Shanghai and Guangzhou and the absolute values of the Pearson correlation coefficients for absolute humidity are larger than those for relative humidity, indicating that the relationship is stronger for absolute humidity than relative humidity. The correlation is not significant in Chengdu.

**Table 6:** Correlation analysis between *R*_0_ and absolute humidity

	Pearson correlation	Significance (2-tailed)	n
Summary	-0.521 ^∗∗^	0.000	84
Beijing	-0.747 ^∗∗^	0.000	21
Shanghai	-0.854 ^∗∗^	0.000	21
Guangzhou	-0.491 ^∗^	0.024	21
Chengdu	-0.166	0.471	21

^∗∗^Correlation is significant at the 0.01 level (2-tailed)

^∗^Correlation is significant at the 0.05 level (2-tailed)

We conducted linear regression on the data of Beijing, Shanghai, Guangzhou as well as the summary of all cities. The linear regression results are presented in Table 7. Replace *a* and *b* in the equation *R*_0_=*a*+*bAH* (where *AH* is absolute humidity) with the corresponding actual values in Table 7, and the correlation between *R*_0_ and absolute humidity can be expressed with a quantitative method.

**Table 7:** Linear regression analysis of absolute humidity to *R*_0_

	*a*	Std. error of *a*	*b*	Std. error of *b*
Summary	2.316	0.031	-0.025	0.005
Beijing	2.412	0.034	-0.055	0.011
Shanghai	2.457	0.034	-0.038	0.005
Guangzhou	2.372	0.087	-0.023	0.009

**Fig. 5:**
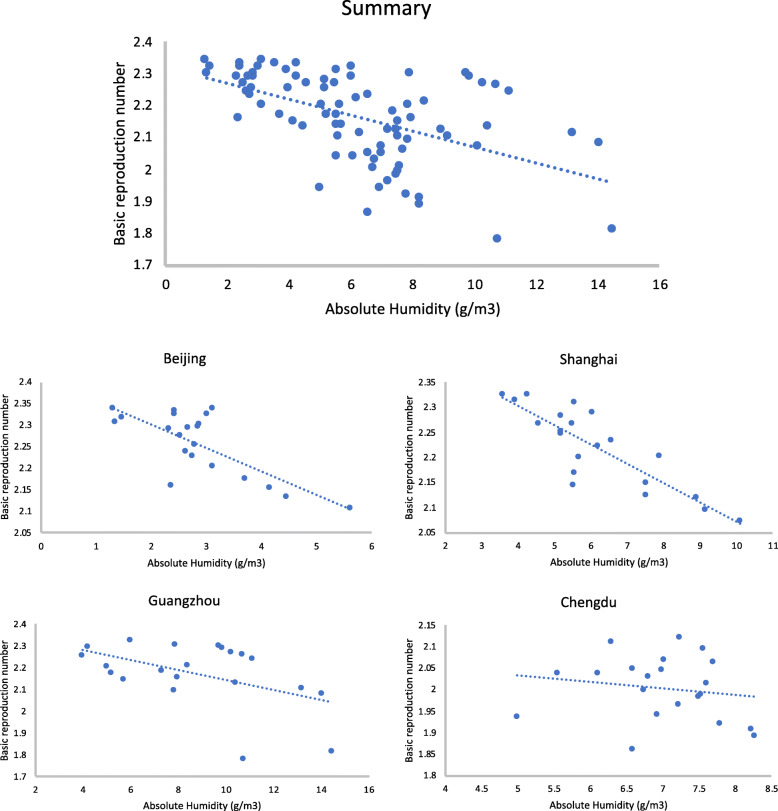
Scatter plot of absolute humidity and basic reproduction number

The scatterplots and corresponding regression lines of absolute humidity and *R*_0_ summarized across all cities and by individual cities are presented in Fig. 5.

### Sensitivity of *R*_0_ to parameters

Substitute the variables in Eqs. (4–7) with *λ*=0.1372 (which is the average *λ* from January 24 to February 13 in Beijing), *T*_*g*_=7.5 and *f*=0.69, and the specific values can be calculated:

8 $$\begin{array}{@{}rcl@{}} R_{0}&=&2.3, \end{array} $$

9) (10) (11 $$\begin{array}{@{}rcl@{}} \frac{\partial R_{0}}{\partial \lambda} &=&10.8,\\ \frac{\partial R_{0}}{\partial T_{g}}&=&0.197,\\ \frac{\partial R_{0}}{\partial f}&=&-0.41. \end{array} $$

When the variables fluctuate within a small range around the given value, *R*_0_ increases as *λ* or *T*_*g*_ increases and decreases as *f* increases. *λ*, *T*_*g*_ and *f* range at the scales of 10^−2^, 10^0^ and 10^−1^ respectively. And the scales of their partial derivatives are 10^1^, 10^−1^ and 10^−1^. Thus the fluctuation scales of *R*_0_ are 10^−1^, 10^−1^ and 10^−2^ corresponding to *λ*, *T*_*g*_ and *f*, which implies that *R*_0_ is more sensitive to *λ* and *T*_*g*_ than *f*. The accuracy of parameters or variables is important for the estimation of the basic reproduction number. As the research on COVID-19 progresses, we can get more precise data and better describe the transmission pattern of the new coronavirus. But the calculation in this paper still makes sense, considering that we focus on relative values instead of absolute values of *R*_0_ in comparison and correlation analysis. Results are reasonable as long as we use the consistent equation and parameters to calculate *R*_0_. By comparison, we can see that the control of COVID-19 is especially urgent in Wuhan and people in other cities should also attach importance to inhibiting the spread of the disease. The vigilance cannot languish until *R*_0_ drops below 1.

## Discussion

### Differences between correlation and causation

In this paper, we discovered the negative correlation between the transmissibility of COVID-19 and temperature and humidity. However, it should be emphasized that correlation is different from causation. According to the Oxford Dictionary, correlation is a connection between two things in which one thing changes as the other does, while causation is the process of one event causing or producing another event. We are not able to infer the causal relationship between two variables solely based on the correlation between them. Correlation is the necessary and insufficient condition of causation. Our results indicated that the transmissibility of COVID-19 was likely to decrease as the temperature and humidity increased. But it did not mean that the increase of temperature or humidity was the cause of the decrease of the transmissibility. We were not able to control other variables in the observation, such as population migration and interventions, which might also affect the transmissibility of COVID-19. So perhaps future work is needed to find out if the changes in temperature or humidity cause the changes in the transmissibility. For example, biological experiments can be conducted by setting the temperature or humidity as the independent variable and the transmissibility of the coronavirus as the dependent variable and controlling other irrelevant variables with the elimination method, constant method, matching method or randomization. Nevertheless, this paper makes sense in terms of confirming that the transmissibility of COVID-19 has a correlation with temperature and humidity and that there is probably a causation relationship between them which deserves further research.

### Effects of temperature and humidity on the transmission of COVID-19

A recent study indicated that temperature and relative humidity held no significant associations with the transmissibility of COVID-19 [40]. It is a very comprehensive and well-conducted research, but we took a step further to take the time series into account by using everyday temperature and humidity. The results show that the overall correlation between *R*_0_ and temperature or humidity is significantly negative, which is consistent with the results of the biological and statistical research on other infectious diseases. It could be explained in several aspects. First, in terms of biological characteristics, a lot of research has confirmed that viruses decay more quickly at high temperature and high humidity [19, 41, 42]. Second, in terms of the transmission media, viruses spread as droplets or aerosols, which maintain large particle sizes at high humidity and thus can settle rapidly or be blocked by masks, nasal cavity, etc [19]. Third, in terms of human immunity, high temperature and high humidity protect the immune organs and benefit people’s health. To sum up, the spread of COVID-19 is likely to weaken at relatively high temperature and humidity and special attention should be paid to the prevention and control of COVID-19 in the coming winter.

As for the correlation in each city, *R*_0_ negatively correlates with both temperature and humidity in Shanghai; *R*_0_ negatively correlates with humidity in Beijing, while the correlation with temperature is not significant; *R*_0_ negatively correlates with absolute humidity in Guangzhou, while the correlation with temperature and relative humidity is not significant; *R*_0_ negatively correlates with temperature in Chengdu, while the correlation with relative humidity is positive and the correlation with absolute humidity is not significant. The deviation of the results may be due to several factors.

First, considering that COVID-19 began in winter, people’s activity and virus transmission mainly occur indoors. In China, the cities north of the Qinling Mountains-Huaihe River Line have central heating indoors in winter. Beijing is north of the Qinling Mountains-Huaihe River Line and Shanghai, Guangzhou, and Chengdu are south of the line. Therefore, the indoor temperature is probably much higher than the outdoor temperature in Beijing, while the indoor temperature may follow a similar pattern as the outdoor temperature in the other three cities. The indoor temperature is probably higher in Beijing than that in Shanghai, Guangzhou, and Chengdu. Although the indoor temperature and the outdoor temperature may have some association, it would be better if we could measure the indoor temperature directly. As for humidity, it has been found that outdoor absolute humidity can be more reliably used as a proxy for indoor exposure compared with relative humidity [43, 44]. Therefore, the correlation between *R*_0_ and absolute humidity may better reveal the situation indoors than relative humidity. Actually, the Pearson correlation coefficients between *R*_0_ and absolute humidity are larger than those between *R*_0_ and relative humidity, proving that the relationship is stronger for absolute humidity than relative humidity.

Second, although Beijing, Shanghai, Guangzhou, and Chengdu are all first-tier cities in China with many similarities like buildings, there are some differences between Chengdu and the other cities that may help explain the positive correlation with relative humidity. Chengdu is located in the southwest of China, the west of Sichuan Basin and the hinterland of Chengdu Plain with a subtropical monsoon humid climate, different from Beijing which has a warm temperate semi-humid continental monsoon climate. The air is more humid in Chengdu than that in Beijing. The climate in Chengdu is similar to the subtropical monsoon climate in Shanghai and Guangzhou, but Chengdu is an inland city while Shanghai and Guangzhou are coastal cities.

Third, the effect of weather on COVID-19 is complicated. The joint distribution between weather and potential confounders should be taken into account. For example, population movement might trigger the transmission of COVID-19 [45]. As for the effects of interventions, we have plotted the time series of temperatures from January 24, 2020 to February 13, 2020 in Beijing, Shanghai, Guangzhou, and Chengdu in Additional file 1: Figure S1. It could be seen from the figure that the temperature kept fluctuating during this period. Considering that the strength of interventions was relatively steady without big fluctuations, which was different from the trends of temperature, perhaps the effects of interventions could be separated from the trends in temperature.

## Conclusions

In this paper, we calculated and compared the basic reproduction number of COVID-19 in 11 major cities in China and analyzed its association with temperature and humidity in Beijing, Shanghai, Guangzhou, and Chengdu to find out the transmissibility of COVID-19 in different cities and its changing trend with the weather. We conclude that the spread of COVID-19 is most violent in Wuhan, Hubei Province and *R*_0_ negatively correlates with temperature, relative humidity, and absolute humidity. Therefore, effective action should be taken to control the transmission of COVID-19 especially in Hubei Province and the transmissibility is predicted to be reduced as the weather warms.

## Supplementary information

**Additional file 1** Figure S1. The time series of temperature in Beijing, Shanghai, Guangzhou and Chengdu.

## Data Availability

Not applicable.

## Abbreviations

COVID-19: Corona virus disease 2019
*R*_0_: Basic reproduction number
SARS: Severe acute respiratory syndrome
MERS: Middle east respiratory syndrome
SEIR: Susceptible-exposed-infectious-recovered
