# Supplementary figures and images for: Transmissibility of COVID-19 in 11 major cities in China and its association with temperature and humidity in Beijing, Shanghai, Guangzhou, and Chengdu
Source: Infect Dis Poverty. 2020 Jul 10;9:87. doi: 10.1186/s40249-020-00708-0 (PMC7348130; doi:10.1186/s40249-020-00708-0)

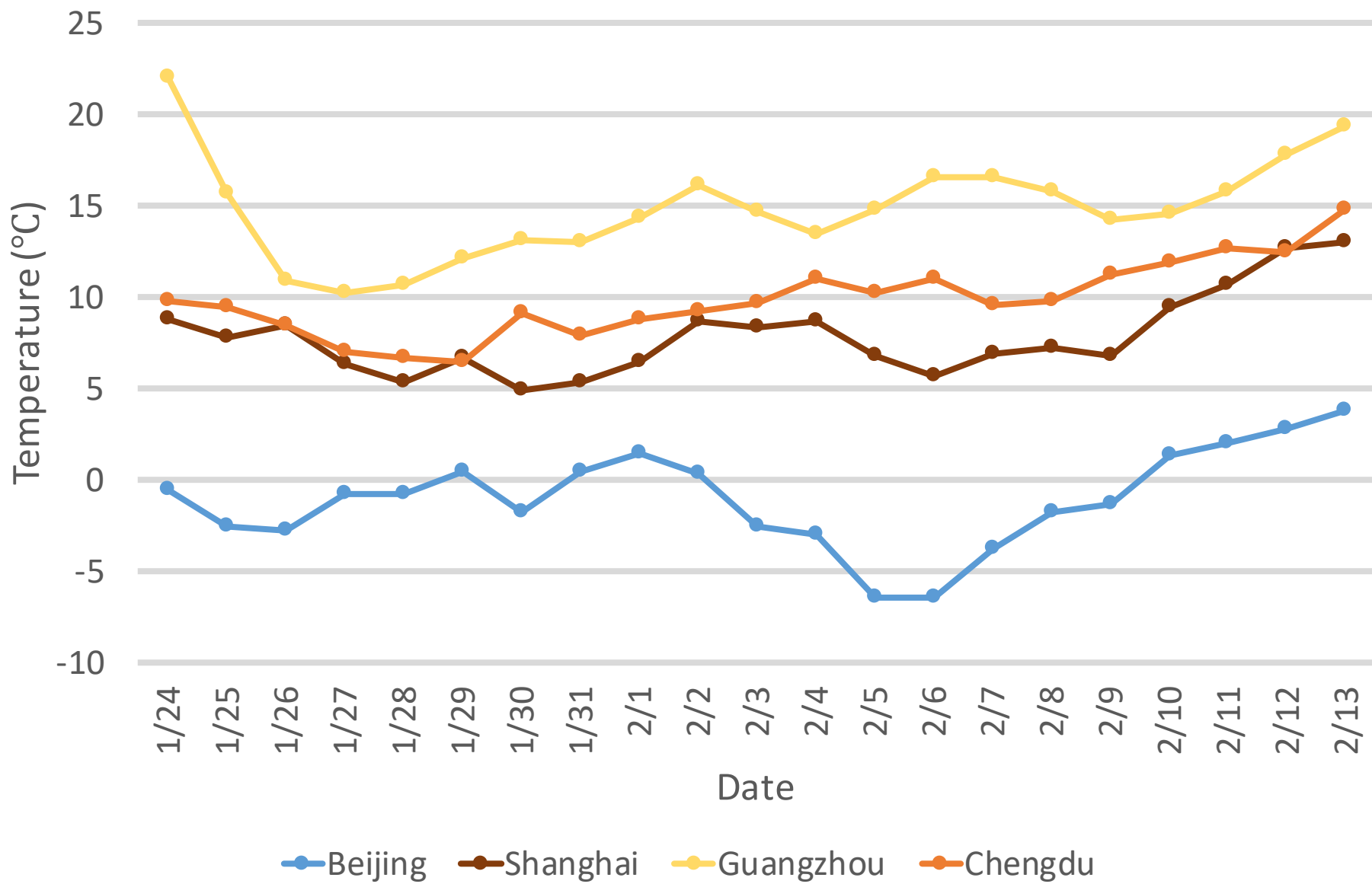

Supplement: Supplementary file 1 — Additional file 1 Figure S1. The time series of temperature in Beijing, Shanghai, Guangzhou and Chengdu. [file 40249_2020_708_MOESM1_ESM.pdf]
